# Functions of behavior change interventions when implementing multi-professional teamwork at an emergency department: a comparative case study

**DOI:** 10.1186/1472-6963-14-218

**Published:** 2014-05-15

**Authors:** Mandus Frykman, Henna Hasson, Åsa Muntlin Athlin, Ulrica von Thiele Schwarz

**Affiliations:** 1 Department of Learning, Informatics, Management and Ethics, Medical Management Centre (MMC), Karolinska Institutet, 171 77 Stockholm, Sweden; 2 Centre for Epidemiology and Community Medicine (CES), Stockholm County Council, P.O. Box 1497, 171 29 Stockholm, Sweden; 3 School of Nursing, University of Adelaide, SA 5005 Adelaide, Australia; 4 Department of Medical Sciences, Uppsala University, Uppsala University Hospital, 751 85 Uppsala, Sweden; 5 Department of Public Health and Caring Sciences, Uppsala University, Box 564, 751 22 Uppsala, Sweden; 6 Department of Emergency Care, Uppsala University Hospital, 751 85 Uppsala, Sweden

**Keywords:** Implementation, Adherence, Fidelity, Operant psychology, DCOM®, Organizational behavior management, Applied behavior analysis, Motivation

## Abstract

**Background:**

While there is strong support for the benefits of working in multi-professional teams in health care, the implementation of multi-professional teamwork is reported to be complex and challenging. Implementation strategies combining multiple behavior change interventions are recommended, but the understanding of how and why the behavior change interventions influence staff behavior is limited. There is a lack of studies focusing on the functions of different behavior change interventions and the mechanisms driving behavior change. In this study, applied behavior analysis is used to analyze the function and impact of different behavior change interventions when implementing multi-professional teamwork.

**Methods:**

A comparative case study design was applied. Two sections of an emergency department implemented multi-professional teamwork involving changes in work processes, aimed at increasing inter-professional collaboration. Behavior change interventions and staff behavior change were studied using observations, interviews and document analysis. Using a hybrid thematic analysis, the behavior change interventions were categorized according to the DCOM® model. The functions of the behavior change interventions were then analyzed using applied behavior analysis.

**Results:**

The two sections used different behavior change interventions, resulting in a large difference in the degree of staff behavior change. The successful section enabled staff performance of teamwork behaviors with a strategy based on ongoing problem-solving and frequent clarification of directions. Managerial feedback initially played an important role in motivating teamwork behaviors. Gradually, as staff started to experience positive outcomes of the intervention, motivation for teamwork behaviors was replaced by positive task-generated feedback.

**Conclusions:**

The functional perspective of applied behavior analysis offers insight into the behavioral mechanisms that describe how and why behavior change interventions influence staff behavior. The analysis demonstrates how enabling behavior change interventions, managerial feedback and task-related feedback interact in their influence on behavior and have complementary functions during different stages of implementation.

## Background

The development of team-based organizations has been strongly recommended for efficiently handling future challenges in health care [1]. This is in line with the breakthrough report “To Err is Human” by the Institute of Medicine, which highlights the importance of interdisciplinary collaboration for further patient safety [2]. Teamwork is recommended as a core component for increasing the value of care [3,4] and in Sweden, the Society of Medicine and the Society of Nursing have emphasized teamwork as a key to improving and securing safe patient care [5]. In addition, empirical studies and a systematic review commissioned by the Swedish Council on Health Technology Assessment [6] have shown that multi-professional teamwork has positive effects on health care processes, patient outcomes and patient safety [7-9]. In sum, there is a strong argument for working in multi-professional teams in health care contexts.

However, the implementation of multi-professional teamwork (denoted teamwork in the following text) has been reported to be complex and challenging. Many prior empirical studies have used education and training of staff teamwork skills as the main implementation strategy for teamwork [10-12]. However, training by itself has often not been enough to create lasting change, and it is suggested that the implementation of teamwork should combine multiple behavior change interventions (BCIs) such as staff training, physical changes to the work environment, and management support [11]. However, although it is known that various BCIs can lead to changes in staff behavior, their effects on staff behavior have varied across studies. Thus, it is not a given that BCIs that work in one setting will work in another setting or at another point in time [13-15]. The mechanisms behind behavior change are unclear, and constitute a barrier for decision makers in their efforts to make optimal use of limited resources for implementation [16]. This calls for studies that increase our understanding of how and why behavior change interventions influence behavior [16-18].

The use of theory and theoretically based behavior change models could shed light on the mechanisms and influence of BCIs [18-20]. Specifically, there has been an interest in psychological theories that focus on the nature of behavior, and thereby offer an understanding of the mechanisms driving behavior change [21,22]. Few prior empirical studies in health care have reported a theoretical rationale for the choice of BCIs [13,15,23,24]. When reported, theoretical models are often based on common sense or intuitive models of behavior. An attempt to make use of theory in implementation is the Theoretical Domains Framework [25]. This framework is based on 33 organizational and psychological theories, integrated into 12 domains relevant to behavior change. Even more detailed is the recently proposed model COM-B, which explicitly describes the functional connections between basic behavioral mechanisms and behavior change interventions [26]. The COM-B describes behavior (B) as a function of three main factors: Competence (C), Opportunity (O) and Motivation (M). It is a comprehensive expert consensus model integrating several theories of behavior change, and is used in the behavior change wheel framework [26].

In line with the COM-B model, applied behavior analysis (ABA) and the underlying theory of operant psychology explain the functional aspects of behavior change [27-29]. ABA suggests that behavior is controlled by two primary factors: antecedents and consequences. Antecedents precede behavior and have an activating function, whereas consequences follow the behavior and determine the probability that it will be repeated in the future. An organization is a system that produces antecedents and consequences in response to behaviors [30]. This suggests that to understand how and why interventions affect practitioners’ behavior, the focus should be on understanding the behavioral contingences provided, removed or changed by the intervention [27,31]. Organizations produce contingencies that either hinder or support desired behaviors and thereby control the probability of the performance of desired behaviors [31,32]. ABA offers an opportunity to study the interrelationships between the different functions and the functional relationship between behavior and the environment. Eccles et al. describe three criteria for a theory to be of value for explaining behavior change in implementation research: (1) The theory should have demonstrated effectiveness in describing and explaining behavior change; (2) Factors explaining behavior should be changeable, i.e. not include unchangeable factors such as gender or age; and (3) The theory should include volitional components, i.e. factors that are in the individual’s power to change, as well as nonvolitional components [19]. In accordance with these criteria ABA has been found efficient in explaining behavior change in various settings, including clinical, educational and organizational contexts [33-35]. The theory is explanatory, includes changeable factors and does not distinguish between volitional and nonvolitional factors.

When applying ABA to organizational behavioral change, a framework called DCOM® is often used [31,36]. The DCOM® framework identifies behavior as a function of four dimensions: *Direction*, *Competence*, *Opportunity* and *Motivation*. Thus, it is similar to the COM-B model described above, the main difference being that the dimension *Direction* is added. This dimension refers to the vertical and horizontal alignment of behavior within the organization; in practice, how well a performer knows what behaviors to perform and how performance is related to the overall goals of the organization. *Direction* is particularly important in understanding organizational change since this, in contrast to individual change, requires individuals to move in the same direction. *Competence* is defined as the skills and knowledge needed for performance. *Opportunity* is the tools, resources and processes provided by the organization that support performance. *Motivation* is the driving force that initiates behavior and, importantly, maintains performance of behavior. Inspired by ABA, the *Motivation* dimension is largely defined by the consequences that reinforce behavior and thereby affect the form, direction and intensity of performance. In contrast to the COM-B model, the dimensions in the DCOM® framework are functionally structured as enabling (*Direction*, *Competence* and *Opportunity*) and maintaining (*Motivation*). Previous research has shown that implementation strategies focusing on predisposing, enabling and reinforcing factors have better results than other strategies [37-39]. Finally, frameworks such as DCOM® or COM-B are based on theoretically derived functions rather than descriptive attributes [26]. This means that the focus is on why an activity leads to behavior change rather than what type of activity it is. For example, training is a type of activity that can have the function of increasing *Competence* and/or clarifying *Direction*. From a functional perspective it is not the activity training per se, but rather its function, that is interesting. In our view, the focus on behavioral functions can further our understanding of how BCIs influence behavior and why they work in one setting but not another.

The aim of this study is to analyze functions of behavior change interventions using applied behavior analysis and the DCOM® model, and to analyze and compare the influence of these interventions on teamwork behaviors at two sections of an emergency department.

## Method

### Study design

This study uses a comparative case study design whereby two sections of the same emergency department (ED) taking different approaches to implementing teamwork are contrasted. A comparative case study design uses the differences between otherwise similar cases to go beyond the in-depth qualitative understanding reached through case studies to also allow the formulation of more general models about the underlying structure that generates the variation between cases [40]. The study is part of a larger research project investigating the outcomes and implementation of teamwork at the ED. Publication within the project includes an analysis of behavior change during the initial phase of implementation [41] and an evaluation of outcomes in terms of lead times [42] and quality of care from a patient’s perspective (Muntlin Athlin A, Farrokhnia N, von Thiele Schwarz U: Teamwork – a way to improve patient perceptions of the quality of care in an emergency department: An intervention study with follow-up, submitted). A study evaluating the sustainability of teamwork is in progress. The study period was 2010–2012.

### Setting

The study was set at the Section of Internal Medicine and the Section of General Surgery of an ED at a university hospital in Sweden, with approximately 55,000 yearly visits (192 per 100,000 inhabitants). A total of approximately 120 nurses (registered nurses (RN) and nursing assistants (NA)) were working at the ED and rotated between the sections. The physicians on call (approximately 180 individuals) were employed within different specialties at the hospital and worked shifts at the ED, ranging from a few shifts each year to a weekly basis.

The traditional way of working included the RN in each section allocating patients to the first available physician. In the proceeding work process, tasks were assigned to any RN. Thus, staff often worked on all patients within the sections. Staff as well as management had limited possibilities to oversee who was working on which patient or what tasks were being done. Thus, patients could be handled by several physicians and RNs who did not necessarily communicate directly with each other.

### The teamwork intervention

The teamwork intervention was initiated by the head of the ED and the senior medical manager at the Section of Internal Medicine. The aim was to increase efficiency of care, defined as adequate and safe care within time limits (set at 4 hours from entering until leaving the ED). More details on the intervention outcomes are published in Muntlin Athlin et al. [42]. The intervention was developed by a work group of staff representatives, management, and two external performance improvement consultants hired by the organization to support the development and implementation of teamwork. Using the performance improvement model described by Braksick [31], the work group pinpointed teamwork behaviors that were then tested, evaluated and refined during two pilots. The intervention that was finally implemented consisted of multi-professional teams including a physician, an RN and an NA. The teams were formed for each shift within the section, and patients were assigned to the teams by a team coordinator. The assigned patient was assessed by the team physician, who made a plan for the patient and divided tasks within the team. Team members performed their tasks together or in parallel, depending on what was considered more efficient. As patients were processed and diagnostic data added, team members continuously met and updated plans, prioritizations and tasks to be done. This way, the whole team was kept updated on the patient’s and team members’ current activities and whereabouts. More details on the content of the intervention are published in Mazzocato et al. [41].

### Implementation of the teamwork

Each of the two sections decided on their implementation strategies separately. As they partly shared staff and location some of the BCIs influenced both sections, but there also were important differences. At the Section of Internal Medicine, the implementation strategy was decided by the work group together with the performance improvement consultants. First, teamwork was tested and developed during pilots in spring 2010, and it was determined that full implementation would start in June 2010. During the summer period, however, the pre-planned reduction of staffing due to summer vacations made teamwork impossible. It was started again in September, which thus is considered the start of implementation in this study. At that time, based on the experience from the spring, the implementation strategy included establishing a change team consisting of the nurse managers and a three-month, full-time change facilitator (physician at the hospital). The change team had the task of systematically developing the intervention and enabling implementation, influenced by the Plan-Do-Study-Act cycle [43] during the three first months, and were coached by the performance improvement consultants biweekly. The overall implementation strategy was based on the assumption that creating organizational opportunities for teamwork and on-the-job training was more important than individual or team-based skill training. At the Section of General Surgery, it was determined that teamwork would be implemented in January 2011. At this time, the senior medical manager allocated a couple of workdays to support the implementation. In addition, the nurse managers (the same persons as at the Section of Internal Medicine) had the task of supporting the implementation. How the BCIs evolved over time is described in more detail in the *Results* section.

### Data collection

**Figure 1:**
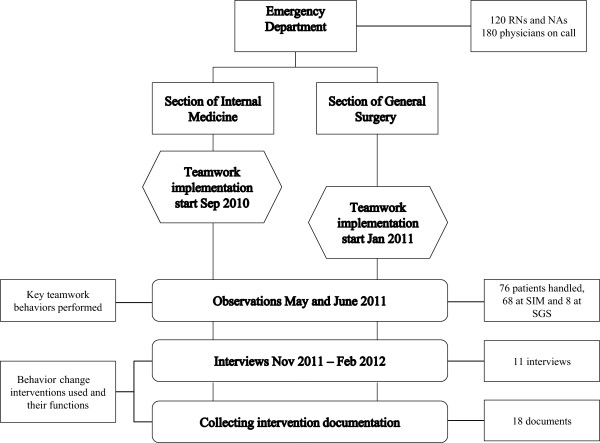
**Data collection overview and timeline.** Description of data collection methods used and a timeline for implementation and data collections.

The data were collected using multiple data collection methods from May 2010 until February 2012. Figure 1 provides an overview of the data collection.

The degree of performed teamwork behavior was evaluated using observations and interviews [44,45]. Observational data were collected in May and June 2011. Three researchers (the first and last authors and an additional research group member) observed staff at both sections. A total of 76 observations (8 at the Section of General Surgery and 68 at the Section of Internal Medicine) were conducted during this period*.* Observations took place during four workdays, and were stopped after saturation had been reached at each section. The unbalanced number of observations at the two sections is due to the absence of teamwork to observe at the Section of General Surgery”. An observation protocol (Additional file 1) focusing on performed teamwork behaviors was used. The following five key team behaviors were used to operationalize the content of the intervention: *Assembling when tasks have been performed, Communicating decision to change plan, Coordinating work, Working parallel, and Communicating the work plan*. One observation represented one team handling one patient, and each behavior was registered as observed or not observed. Questions were asked to clarify behaviors.

A total of 11 semi-structured interviews were conducted by the first author between October 2011 and February 2012*.* A general interview guide (Additional file 2) based on the DCOM® model was created by the first and last authors, and covered the following themes: Perceived intervention changes and outcomes, Program theory, Description of activities and behaviors during the different phases of the implementation, Challenges, How challenges were handled, and Challenges for sustaining the change. A purposive selection criterion was used, and four respondents were selected for the interviews by the researchers based on their central role in the implementation process. Respondents included the senior medical manager at the Section of Internal Medicine who had initiated teamwork at the ED, a change facilitator (Section of Internal Medicine), a nurse manager (working at both sections) and the senior medical manager at the Section of General Surgery. Snowball sampling was used to identify additional respondents [46]. This resulted in seven further key persons, both managers and staff, being identified and interviewed. The respondents were informed that their participation was voluntary and that data would be handled in a confidential manner. A consent form was signed. Each interview lasted 30–90 minutes. All interviews were audio recorded and transcribed verbatim.

#### Documentation

All documentation related to the intervention and implementation at the two sections was collected. Relevant documentation was identified primarily during the interviews and through the performance improvement consultants. The collected documents (n = 18) included information material about the intervention, i.e. presentations to staff and a description of workflow (n = 6), descriptions of the intervention from the performance improvement consultants (n = 4), the ED weekly information sheet (n = 2), teamwork checklists (n = 3) and role descriptions (n = 3). Documents are described in more detail in Additional file 3. Generally, the documents were designed for the Section of Internal Medicine (as they were the initiators and the first to implement teamwork) with the intention that they would also be applied at the Section of General Surgery.

### Ethical approval

Ethical approval was granted by the regional ethical review board in Uppsala (Dossier number 2010/170).

### Data analysis

Interview data were independently analyzed by the first and last authors, using a hybrid thematic analysis [47] and following the step-by-step approach described by Shilling [48]. The hybrid thematic analysis integrates a deductive, theory-driven approach with an inductive, data-driven approach [47]. A category system based on the DCOM® model was defined before the analysis and was used as a guide for the analysis [49]. Raw data were condensed into text components, the smallest size being sentences and the largest complete sections of text, interview by interview. The text components were categorized according to the category system. Categorized text components were then condensed node by node a second time into shorter sentences [48]. Recurring themes were identified and compared to documentation for validation. This resulted in two final coded protocols with staff perceptions of performed teamwork behaviors and BCIs categorized according to the DCOM® model. Data were organized using NVivo 10, a qualitative data analysis software program.

To test inter-rater reliability, the other two authors independently compared the final two codings. A percentage of item correspondence between the two codings was calculated for each DCOM® element and description of teamwork behaviors. The comparison of the codings was then discussed with the first and last authors. During the discussion some of the disagreements were clarified and therefore solved, and final inter-rater reliability percentages were calculated. These ranged between 75% and 100% agreement for the four dimensions of the DCOM® model. The result of the analysis was corroborated in discussions with the external performance improvement consultants and representatives of the ED. As a final step, documents were used to triangulate the interviews and observation data. Documents were studied to identify deviations within them and in relation to the interviews. Triangulation was performed by the first author, and all documentation described above was used.

## Results

### Degree of performed teamwork behavior

**Figure 2:**
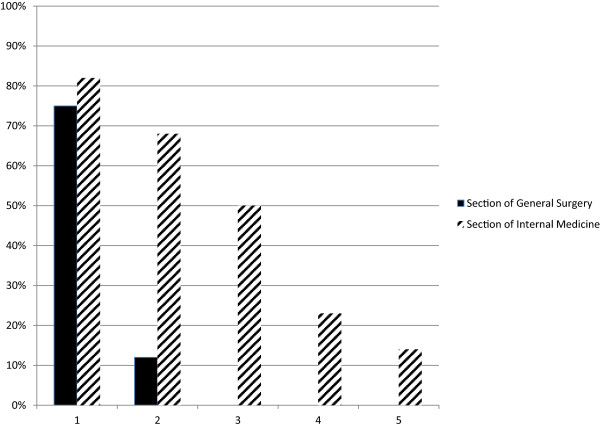
**Observed teamwork behaviors.** Percentage of cases observed where one, two, three, four or five of maximum five teamwork behaviors were identified.

The results showed that the teamwork behaviors were more frequent at the Section of Internal Medicine compared to the Section of General Surgery. Figure 2 describes accumulated percentages of observed teamwork behaviors for each section. At 50% of the observations at least three teamwork behaviors were observed at the Section of Internal Medicine, compared to 0% at the Section of General Surgery. At the Section of Internal Medicine, each teamwork behavior was observed during at least 40% of the observations. At the Section of General Surgery *Communicating the work plan* was observed at 75% of observations and only one other behavior, *Working parallel*, was observed (and only on one occasion). In fact, the observations at the Section of General Surgery were stopped after eight observations because of the lack of teamwork to observe; thus the low number of observations.

Interviews with staff and management representatives at the Section of Internal Medicine supported the observation findings, and gave a congruent and detailed description of teamwork behaviors. They described the most important elements of teamwork as: team members assembling and starting the shift together; distribution of tasks among team members; recurring gatherings in team rooms; and ongoing communication and coordination of tasks during the day between team members. Overall, this description of teamwork corresponds well with the key teamwork behaviors described in the documentation. The exception was team members assembling and starting the shift together, which was not part of the protocol although it was considered an important element of teamwork, as the observation protocol was structured around each patient case rather than the team as such. Respondents’ descriptions of teamwork coverage among staff were congruent, and staff deviating from teamwork was described as very infrequent.

At the Section of General Surgery, staff and management were less detailed in their descriptions of teamwork, and overall gave an incongruent description of its important elements. Teamwork coverage among staff was divergent, ranging from full to poor. Teamwork behaviors were reported to vary depending on who was working, mainly referring to the team physician. The majority of respondents at the Section of General Surgery described that teamwork was performed to some extent for about a month after its start and then faded out.

#### Behavior-changing interventions and their functions

**Figure 3:**
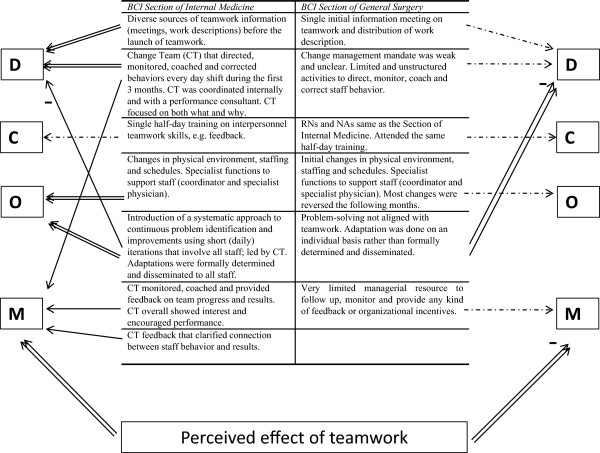
**Categorization of main behavior change interventions using the DCOM® dimensions.** Description of main behavior change interventions (BCIs) identified at each section. The arrows describe the BCI influence on the dimensions *Direction* (D), *Competence* (C), *Opportunity* (O) and *Motivation* (M). A dotted arrow indicates a weak influence on the dimension; a single-lined arrow indicates a medium influence; and a double-lined arrow indicates a strong influence. A minus sign at the end of the arrow indicates a negative influence on the dimension. The effect of teamwork perceived by staff is an implicit result of BCIs rather than a BCI in itself, and is thereby categorized in a separate box.

Figure 3 describes the main BCIs at the two sections, categorized according to DCOM®. Overall, more BCIs were reported at the Section of Internal Medicine compared to the Section of General Surgery. Below, the BCIs and their functions at the two sections are described.

### Section of Internal Medicine

The teamwork intervention was introduced successively during spring 2010 by the senior management, managers and external performance improvement consultants. Communication was coordinated during introduction, explicitly involving senior management, and had the function of clarifying *Direction* early in implementation. During the initial implementation phase, BCIs providing *Direction* and *Opportunity* by the change team were particularly frequent. Two members of the change team were always present during day shifts, and actively supported staff in their teamwork efforts. The change facilitator had full authority, which is supported by both interviews and documentation, and acted to direct staff and implement necessary changes to enable teamwork. Staff and management gave a coherent description of what was expected from them and why, indicating an overall strong *Direction*. This was further enhanced by the fact that key actors, including managers at all levels, were vertically aligned in terms of pointing out the *Direction*. The highly structured teamwork process, i.e. stringent *Direction*, was perceived by some as initially demotivating as autonomy was reduced. Thus, some of the activities had dual functions. Similarly, active monitoring and feedback from the change team were BCIs that primarily had a directional function, but the interest and support that came with these activities were also perceived as motivational.

Hardly any BCI had the primary function of increasing *Competence*, which can be understood in staff descriptions of all staff having the skills necessary to perform teamwork behaviors. However, some described that professional proficiency affected team functioning. For example, it was hard for the teams to synchronize work tasks when an inexperienced physician had trouble keeping up with experienced nurses and vice versa.

In terms of *Opportunity*, staff described that the physical environment and staff resources were adapted so that teamwork was satisfactorily supported. They particularly highlighted the importance of problem-solving, which they described as engaging and productive. However, the initial frequent problem-solving also meant that there were frequent changes to the teamwork process and the physical environment. This made it hard for staff to keep up with the changes, as they perceived that they got new directives “each time” they started a new shift. Thus, although the changes overall were perceived as necessary and being clearly communicated, for example through the weekly information sheet, the frequent problem-solving initially had a negative impact on *Direction*. Thus, the BCIs primarily associated with problem-solving had a negative secondary function. Problem-solving was a time-consuming BCI that went on for three months.

One of the few motivational BCIs identified was the members of the change team meeting staff at the end of each shift to discuss team performance and barriers to performance, following a pre-defined structure as described in the debriefing checklist. Members of the change team also provided feedback on the number of patients handled by the team during the shift. This helped clarify the connection between performance and results. Thus, the change team provided ongoing coaching and support to the staff. Also, the attention and interest of the change team was perceived as motivational by staff. However, the most important motivational factor was that the staff experienced personally valued results of teamwork. This included: working with fewer patients, thus enabling a better overview and making it possible to see the results of one’s own work; being able to give more precise information to patients to relieve their anxiety; perceiving more efficient delivery of care; and less cognitive load and, overall, less stress. Importantly, these results were not evident until a few months after the teamwork had initially been implemented. Before this, particularly during the first three to four weeks of teamwork, staff even experienced some negative results such as confusion, a slower workflow and frustration with practical barriers.

### Section of General Surgery

Teamwork was introduced at the Section of General Surgery five months after the Section of Internal Medicine. Thus, RNs and NAs already had a clear sense of *Direction,* whereas teamwork was new and *Direction* less clear for a majority of the physicians at the surgical section. The implementation was introduced by the nurse educator and managed by the nurse managers and senior medical manager at the Section of General Surgery. However, their mandate from senior surgical management to carry out the implementation was perceived by staff as unclear. This was especially important as many of the physicians at the Section of General Surgery were described by the respondents as experienced surgeons, not accustomed to taking orders from RNs or the senior medical manager at the ED. The time allocated for managing the implementation was a couple of extra shifts during the first weeks of initial implementation. There was no structured coordination regarding the implementation between the senior medical manager and the nurse managers. In contrast to the Section of Internal Medicine, staff and management descriptions of teamwork aim and expected key teamwork behaviors at the Section of General Surgery were inconsistent and somewhat contradictory. Managers were not perceived as vertically aligned, and the limited monitoring, correction and feedback performed by management were not coherent. Staff and management trust in teamwork as a work process to achieve the department’s main goals was incoherent. After about a month, deviations from teamwork had become obvious and as these remained uncorrected this further weakened the already unclear *Direction*.

As in the Section of Internal Medicine, hardly any BCIs had the primary function of increasing *Competence*. Staff also perceived that they had the skills necessary to perform teamwork behaviors. However, limited professional proficiency of inexperienced staff affected team functioning negatively in terms of disrupting the workflow for the other team members.

Initial changes to the physical environment, schedules and specialist work descriptions were made to increase *Opportunity*. As time passed and additional barriers were identified these initial changes, made to enable teamwork, were reversed to some extent. The problem-solving process was perceived as unsystematic and lacking a common goal, i.e. sometimes performed in a way that hindered teamwork but prioritized other work processes such as the supervision of interns. A secondary function of the problem-solving process was that it made *Direction* increasingly unclear, i.e. implicitly supporting changes that did not prioritize teamwork. *Opportunity*, thus, did not enable teamwork.

Overall, management interest and support were perceived as limited and unsystematic, providing sparse *Motivation* for staff. The initial results of teamwork were primarily perceived as negative and frustrating, i.e. involving confusing processes and a slower workflow. A smaller group of the respondents described a shift towards perceiving positive valued results of teamwork after about a month, i.e. faster workflow and a less stressful work environment. The ones describing positive results were primarily staff who had experience of teamwork from working at the Section of Internal Medicine. *Motivation* derived from social interaction was described as contradictory. This means that among themselves, staff members were as likely to be criticized for performing teamwork behaviors as for not doing so, depending on the source of the communication. Criticism for performing teamwork was said to be related to the level of commitment to implement teamwork. Overall, *Motivation* was weak, lacked reinforcing consequences and was dominated by negative perceptions of teamwork consequences.

## Discussion

The two ED sections differed greatly in the degree of performed teamwork behaviors. They also differed in the behavior change interventions that were used. At the Section of Internal Medicine, where teamwork was implemented to a higher degree, BCIs focusing on clarifying *Direction* and providing *Opportunity* to perform teamwork behaviors were primarily used. These also had motivating functions. Motivational BCIs played an important but temporary role during the initial implementation. Experiences of positive results of teamwork, e.g. task-related feedback, were more important for long-term motivation and maintained behavior change. The findings are discussed in more detail below, together with reflection on the usefulness of the DCOM® model.

The two sections used different BCIs when implementing teamwork. The functional analysis of the BCIs showed that the enabling dimensions *Direction* and *Opportunity* were the most important in supporting the implementation of teamwork at the Section of Internal Medicine. During program installation, directional BCIs involved influential messengers at different managerial levels, which has been highlighted as essential in prior studies as well [50,51]. The intensity of directional BCIs increased substantially during initial implementation. This was important for a number of reasons. For instance, the physicians working at the Section of Internal Medicine changed regularly and were not familiar with all the routines. In addition, teamwork was systematically adapted during the installation phase, and thus the adjustments needed to be communicated to staff. The directional BCIs also entailed management giving correctional feedback on team behaviors. This corrective action offered few options for alternative behaviors. The Section of General Surgery used fewer influential messengers when introducing teamwork. Upper management was hardly involved, and did not provide *Direction* or create *Opportunity*. Directional BCIs were not systematically repeated during initial implementation, and the content of the directional BCIs was not congruent; i.e. staff received different, often conflicting, directions. This resulted in a wide range of individual interpretations of teamwork behaviors. In addition, staff did not agree on teamwork as a means to achieve department goals. Prior studies emphasize the importance of creating positive attitudes and anticipation regarding the results of change [52,53]. From a theoretical stand point this created an initial motivation to engage in key behaviors by increasing valence, i.e. how you value the result of a change. From a functional perspective, real-life experience of consequences is the strongest determinant of behavior change [27], suggesting that anticipated consequences are important during the initial stage of implementation but that the effect diminishes as it is replaced by real-life experience of behavioral consequences. This is also known as operant conditioning and describes the well documented psychological process whereby the effect of antecedents, i.e. anticipated consequences, on behavior is altered as a function of real-life consequences [29]. Social cognitive research also supports this in that it emphasizes experience as the strongest determinant of self-efficacy [54], an important predictor of behavior change. Managerial consequences, such as feedback and managers showing interest, are also known to influence staff behavior [55,56]. In all, this suggests that directional BCIs have an important but short-term motivational influence on behavior change, and need to be supplemented with motivational BCIs to create sustainable change.

The *Competence* dimension was not described as important for implementing teamwork. However, staff at the Section of Internal Medicine most certainly developed skills in terms of learning the new work processes and roles during the first months of on-the-job training. That is, the low importance of *Competence* does not mean that no skills were developed. Rather, it might reflect that (1) the brief training focused on interpersonal competence that staff experienced that they already had, and (2) the learning of new work processes and roles was perceived as a natural part of adapting to the new work process.

*Opportunity*, i.e. the tools, resources and processes provided by the organization, at the Section of Internal Medicine consisted of two parts. The first involved changes in staff resources and room allocations based on a pre-implementation analysis of barriers, and was performed mainly during program installation. The second part took place during initial implementation, and involved ongoing problem-solving. The complexity of the organization made it difficult to foresee all obstacles. This meant that creating *Opportunity* before implementation start was important, but not sufficient, for handling barriers to performance. With the functional perspective of ABA, the organizational barriers initially provided punishing consequences in response to key teamwork behaviors and thereby decreased the probability that the behaviors would be repeated [27-29]. The punishing consequences included, for instance, a slower work pace and frustration with practical barriers. With the absence of systematic problem-solving at the Section of General Surgery, staff did not deal with the barriers, and as punishing consequences accumulated staff reverted to the traditional work process as a way to avoid the punishing consequences associated with teamwork. The functional analysis of behavioral consequences suggests a way to understand the underlying mechanisms of behavior change and how the short-term adoption, but long-term desertion, of key behaviors might be understood. This is an important contribution to understanding sustainable implementation, which has been cited as an area in need of further research [57]. In summary, the ongoing provision of problem-solving activities at the Section of Internal Medicine was important in enabling teamwork behaviors as it changed important factors in the organizational context. Thus, problem-solving was used to improve the fit between the intervention and the organizational context, thereby increasing the probability of reinforcing consequences. This is the core of tailored interventions [58]. However, tailored interventions often focus on the pre-installation identification of barriers. The findings from this study suggest that this may not be enough in interventions involving complex change. Instead, an ongoing identification and handling of barriers to change may be necessary. This type of continuous improvement is the core of many improvement models, such as Kaizen [59] and PDSA [43]. However, it seems that ongoing problem-solving needs to be combined with concurrent directional BCIs that communicate adaptations to the program.

Staff motivation to change behavior at the Section of Internal Medicine was primarily a function of task-generated feedback from engaging in teamwork behaviors [60]. As teamwork was enabled and barriers to performance were removed, staff experienced both fewer punishing consequences and more positive and valued consequences when engaging in teamwork behaviors. That is, teamwork behavior was conditioned by real-life consequences. This means that *Motivation* was an implicit result of enabling BCIs in combination with staff valuing the task-generated feedback that came from engaging in teamwork behaviors. Task-related feedback involving direct response from multiple sources, including patients, has previously been shown to be related to high work motivation [61]. At the Section of General Surgery staff did not experience positive task-generated feedback, as teamwork behaviors were not sufficiently enabled and sustained. Overall, task-generated feedback, which comes naturally as a response to behavior, is emphasized as an important motivational factor in many motivational theories [61-65]. It is intrinsic rather than extrinsic in character, and is thereby considered to have a stronger motivational effect [62,66]. This case is an example of how task-related feedback is delayed and not fully distributed during the first months of implementation. Basically, this was because it took time to get the new work practices to function well enough to create positive task-related feedback. However, management feedback and small daily improvements were important motivational BCIs during the two first months of implementation, and bridged the motivational gap before task-related feedback was fully enabled. It is known that managerial feedback can increase motivation [55,60], and that managerial activities, e.g. feedback, problem-solving etc., are especially important for new teams [67]. Thus, this study suggests that controllable BCIs, such as management feedback, encouragement and problem-solving, may have a more important motivational function during initial implementation but less so later, given that the change involves task-generated feedback. Another possible explanation for motivation during the first months of implementation is that the experience of small daily improvements had a motivating effect during this period. This is in line with operant psychology [29] and theories on work motivation, such as control theory and social cognitive theory [54,68].

Practically, these findings describe how organizations can optimize the implementation of new ways of working by choosing BCIs based on which function (*Direction*, *Opportunity*, *Competence* or *Motivation*) is the most important.

### Methodological discussion

At the Section of Internal Medicine, the fidelity to the teamwork intervention was described by the staff as sufficient despite the fact that the number of teamwork behaviors performed in each patient case varied. This may reflect that not all team behaviors were applicable to all patients at all times, such as uncomplicated cases that only required attention from the physician. It also reflects that some key behaviors are contingent on specific events; for example, the teamwork behavior *Communicating decision to change plan* can only take place once there has been a change in plans. This is in line with a prior study showing how some intervention components need to be customized to each patient [45], and highlights the need to allow local adaptation. The Replicating Effective Programs (REP) framework suggests that the core elements of an intervention should be standardized, but that the mechanism by which they are operationalized can be changed to allow flexibility in implementation [69]. In other words, the judgment of which components are to be delivered to each patient should be made by staff as long as this is in line with the overall aim, i.e. *Direction*. This suggests that the measurement of actual behavior change when implementing methods consisting of multiple components should carefully consider which behaviors are necessary for determining fidelity levels.

This study involved two sections that implemented the same work processes in very similar settings [70]. The main differences in the implementation procedures were different management, different physicians and start-up five months apart. This gives us favorable conditions for comparing the impact of the different behavior change interventions used, in line with comparative case study methodology [40]. External validity of the study might be limited due to the Swedish model depending on physicians on call rather than full-time specialists in emergency care. This could have an effect on the importance of the *Direction* dimension. On the other hand, external validity is strengthened as the BCIs used are common techniques when implementing teamwork in other settings. Thus, the function of the dimensions should be relevant for other organizations. Rather than developing a new model, this study is based on basic psychological science and well-established models for behavior change. Using established theories and models contributes to the accumulation of knowledge.

A number of validity criteria for trustworthiness need to be highlighted and discussed [71]. To show credibility, well established qualitative methods were used and the whole research team participated in the data analysis. The study is part of a larger longitudinal intervention project; thus, the researchers were familiar with the context and the staff. The sampling procedure was purposive, using informants with a central role in the implementation. The triangulation, using observations, interviews and documentation, was useful in establishing confidence in the truth of the data. Analysis of qualitative data was performed by researchers with at least five years’ experience with the models used, which is considered a strength as the theory-driven approach used is regarded as especially sensitive to the experience of the researcher [49]. Credibility was limited by the number of interviews. Also, the complex ED environment with a high number of staff members (some temporary) as well as high tempo and workload sometimes made observation challenging. This was handled partly by using many observers, who continuously discussed their observations, and through the large number of observations. Regarding dependability, the research design is well documented and the team regularly reflected upon the process. To address transferability to other settings or groups, awareness of the clinical context and culture is necessary. Some of the BCIs’ functions, e.g. problem-solving, might be more important in complex contexts.

### Implications for practice

Teamwork is a promising intervention to improve health care. However, in practice, both teamwork and its implementation may look very different in different settings. The most important lesson from this study is not a specific teamwork intervention. Rather, this study highlights how teamwork can be implemented and suggests that clear *Direction* (i.e. engaged management that is specific regarding both why and how teamwork should be performed, alignment between teamwork and other processes and initiatives) and allocation of resources for ongoing problem-solving and adaptation are important ingredients for effective implementation of teamwork, and possibly other complex changes. This is especially important in organizations with a rapidly and continuously changing context. Also, when implementing interventions that are not immediately rewarding for staff, it is important that management or a change team actively support and motivate the staff.

## Conclusions

The functional perspective of applied behavior analysis offers insight into the behavioral mechanisms that describe how and why BCIs influence staff behavior. This adds important information to the understanding of successful implementation processes. In this study ongoing problem-solving (*Opportunity*), in combination with a clear and coordinated *Direction*, enabled teamwork during the initial implementation phase. In combination with motivational BCIs such as managerial feedback, this was initially important for starting up and supporting the behavior change before staff could experience task-generated feedback. Once task-generated feedback was established, the importance of other motivational BCIs decreased. Thus, different motivational BCIs, such as task-generated feedback on the one hand and managerial feedback on the other, can have complementary functions in motivating behavior change during different stages of implementation. Based on a theoretical model, our analysis demonstrates how enabling interventions, managerial feedback and task-related feedback interact in their influence on behavior and have complementary functions during different stages of implementation.

## Competing interests

The authors declare that they have no competing interests.

## Authors’ contributions

The present study is part of a research project investigating teamwork at an emergency department (the TEPPP study). The authors’ contributions are as follows: AMA and UvTS initiated the TEPPP project and secured funding and ethical approval for the project. MF, HH, AMA and UvTS jointly conceived the idea for this study. MF and UvTS performed observations, and MF the interviews and collection of documents. MF performed the literature search in collaboration with HH. MF and UvTS analyzed the data, HH and AMA reviewed the analyses, and MF wrote the initial draft of the manuscript. All authors contributed to the drafting of the manuscript and provided critical revision, and have read and approved the final manuscript.

## Pre-publication history

The pre-publication history for this paper can be accessed here:

http://www.biomedcentral.com/1472-6963/14/218/prepub

## Supplementary Material

Additional file 1 Observation protocol: teamwork. Click here for file

Additional file 2 General interview guide. Click here for file

Additional file 3 Intervention documentation. Click here for file
