# Supplementary material for: Functions of behavior change interventions when implementing multi-professional teamwork at an emergency department: a comparative case study
Source: BMC Health Serv Res. 2014 May 15;14:218. doi: 10.1186/1472-6963-14-218 (PMC4050988; doi:10.1186/1472-6963-14-218)
Supplement: Additional file 1 — Observation protocol: teamwork. [file 1472-6963-14-218-S1.pdf]

Observation protocol: teamwork

Observations of the team's work are conducted from a patient perspective – how is the patient handled by the team?

DATE:

OBSERVER:

Team composition: Emergency physician ☐ RN ☐ RN ☐ NA ☐ NA ☐ Specialist ☐ Resident physician

Observation conducted between the times ________ and ____________________

Summary:

Total number of patients the team has been responsible for during the observation time:

____

Number of these who have been handled, completely or partially, by someone not on the team:

____

Time of day	*Number of patients – monitored*	*Number of patients – not seen*	*Other*
-			
-			
-			
-			

In total, to what degree have the team members known what the others on the team did during the day?

About the day - other:

Patient data

Process code (color code): Male ☐ Female ☐ Year of birth ________Reason for visit:_____________________

Patient arrives at: __________________________ ...to exam room:____________

Patient finished at ED: _______________ Patient leaves emergency department: _______________

Is the patient arriving from *reception* ☐ *emergency room* ☐ *from the shift before*☐ *other, namely* ____________________?

Already triaged *Yes* ☐ *No* ☐ By whom?____

RN's triage efficient? (if possible to observe)

- *Reason for visit – why today and not yesterday?* ☐ Yes ☐ No
- *When did the problems start? How did they start?* ☐ Yes ☐ No
- *Do you have any contagious infections?* ☐ Yes ☐ No

Who takes the first history?

Physician ☐ RN ☐
How long after the patient is placed in the exam room? ____________________________

Are the physician and RN together in the room? *Yes, the entire time* ☐ *Yes, at first* ☐ *No* ☐

If "Yes, at first" : How long does the RN stay in the room?_____________________________

Why did the RN leave the room? What happened immediately before and immediately after?

Physician's history taking efficient?

Reason for visit – why today and not yesterday? ☐ Yes ☐ No

When did the problems start? How did they start? ☐ Yes☐ No

Do you have any contagious infections? ☐ Yes ☐ No

Diseases? ☐ Yes ☐ No

Medicines? ☐ Yes ☐ No

Quick plan?

How quickly does the physician have a plan for the patient?

____

Physician – team leader – explains plan to RN	☐ Yes ☐ No
Physician – team leader – explains plan to patient	☐ Yes ☐ No
Physician – asks the rest of the team about their observations	☐ Yes ☐ No
RN's spontaneous comments? Questions? Explanations?	☐ Yes ☐ No
Team members double-check information ____________(counts)	☐ Yes ☐ No

How long does the plan cover? *the next step* ☐ *prel diagnosis* ☐ *prel treatment*☐

Quick implementation of plan?

Time between prescription/examination (by physician) and treatment/implementation (by RN): ____________ min

Does the team cooperate and consult with each other regarding the patient?

Do the team members work with the patient in parallel?	☐ Yes ☐ No
Do the team members coordinate the work?	☐ Yes ☐ No
When a decision is made to change the plan, do the team members communicate this?	☐ Yes ☐ No

How often do the team members wait for each other or look for each other?

Does the team complete the work with the patient according to plan?

Do the team members communicate about the decision to discharge the patient?	☐ Yes ☐ No
Do the team members gather in the team room when finished working with the patient?	☐ Yes ☐ No
Are possible deviations from the plan intentional/planned?	☐ Yes ☐ No

What factors hinder a quick handling of patients?
