# Supplementary material for: Functions of behavior change interventions when implementing multi-professional teamwork at an emergency department: a comparative case study
Source: BMC Health Serv Res. 2014 May 15;14:218. doi: 10.1186/1472-6963-14-218 (PMC4050988; doi:10.1186/1472-6963-14-218)
Supplement: Additional file 2 — General interview guide. [file 1472-6963-14-218-S2.pdf]

General interview guide

The following is the general interview guide, containing questions for the Section of Internal Medicine and Section of General Surgery as well as the managers and staff in the respective sections. At the time of interview, the questions were adapted based on the headings and instructions below.

Explain question:

Understand the implementation of teamwork through comparison of General Surgery and Internal Medicine.

Outcome - current

1. What are the biggest changes you have experienced? (as a result of the measures taken)
    a. What characterized the work before the implementation of teamwork?

2. What results were expected? (relate to program theory)

3. What results can be observed for a) patients, b) staff?

To staff:

Were you here before? If not, what have you heard about the time before the teamwork?

Differences between General Surgery and Internal Medicine

Exploration and adoption (program theory):

Medical – During fall 2009, the decision was made to implement teamwork.

1. When did the decision process that ultimately resulted in teamwork begin?

2. What did you want to achieve?
    a. What was the main goal/aim of implementing teamwork (were there more?)?
    b. In what way did they/you believe teamwork would contribute to the goal/aim?

3. Were both General Surgery and Internal Medicine included from the beginning? Were there differences in the reasoning and conditions for General Surgery and Internal Medicine?

4. Has/have the development work/ideas been developed, refined, changed during this time?
    a. We know this partially. The leader track was removed and the focus moved to the team in spring 2010.

Additional questions:

1. What do you mean by teamwork?

2. Were there alternatives to teamwork?

3. If so, why did the result end up being teamwork?

Program installation

NOTE: To be asked at General Surgery also!!!

Between the decision to implement teamwork and its actual introduction in summer 2010...

1. What contributions were made (and by whom) to prepare for the implementation of teamwork?
    a. *Were the contributions at General Surgery and Internal Medicine different?*

2. What were the challenges you expected?

3. What was important during this period?
    a. What characterized the work?

Additional questions:

1. When and how was teamwork presented to the staff?

2. Were barriers/challenges analyzed?

3. If so, what were they?

4. To what degree did you experience that staff members were motivated or not?

5. The teamwork was refined by having a ”test group” systematically test and evaluate it. Expand on this.

6. How was the teamwork concretized?

To staff:

1. When did you first hear that teamwork was being planned?

2. What did you think about it? What barriers/possibilities?

3. Looking back, what contributions for implementing the teamwork were important for you and your coworkers to be able to start working in teams?
    a. What characterized the work during this period?

Initial implementation

Medical – At Internal Medicine, the teamwork was implemented on two occasions: partly in May/June 2010, and then after summer 2010...

Surgical – At General Surgery, the teamwork was implemented later than at Internal Medicine...

1. How did the actual transition from traditional work to teamwork happen?

2. What contributions aimed at supporting and facilitating teamwork initially (the first 6 months)?

3. Which actors were especially active/participatory in the process?

4. How were you (management) able to follow up how it was going?

Additional questions:

1. Did the fact that the teamwork was implemented twice have any effect?

2. What barriers/possibilities became clear upon implementation?

To staff:

NOTE – Distinguish between the sections! Ask which one the interviewee means!

1. What do you remember from the first time the teamwork was implemented?

2. What do you yourself feel helped you change your behavior and start working within a team?

3. What parts of the teamwork were easier/harder to start doing? Why? (Were there no interventions?)

4. Are there differences in the teamwork between Internal Medicine and General Surgery? Expand on this – who and what are the differences due to?

Innovation

1. What have you learned about teamwork?

2. Has the way you work in teams developed in any way?

3. How has this development happened?

4. Have you identified barriers to efficient teamwork during the year?

5. How have you handled these barriers?

Surgical, cont'd from above...

1. What are General Surgery's future plans for teamwork?

2. What have you learned from Internal Medicine that you want to use in a new attempt at implementing teamwork at General Surgery?

To staff:

1. What have you learned about teamwork during the year you have worked in teams? What changes have been made?

2. How have the changes been made?

3. What during this year has made it easier for you to work within a team?

4. What has made it difficult?

Full implementation

1. What are your greatest challenges for this to be maintained and be a value-adding way of working in the future?

Other questions

1. What ”outside” factors/occurrences have affected <u>the process</u>? When and how have they had an effect?
    a. *Different levels: national, county council, hospital*
    b. *Different aspect: organizational, management changes, other development projects, savings requirements, new IT system, etc.*
    c. *What has the interaction between different system levels looked like over time?*

2. How have the key actors worked with/affected the surroundings?
    a. *Acted upward/outward to create the right conditions, or inward to make it happen?*

3. What have the premises’/department’s conditions been? In what way have these affected the course of events?
    a. *For instance support, resistance, resources, competence, experience of previous changes, culture, etc.*
