# Supplementary material for: Functions of behavior change interventions when implementing multi-professional teamwork at an emergency department: a comparative case study
Source: BMC Health Serv Res. 2014 May 15;14:218. doi: 10.1186/1472-6963-14-218 (PMC4050988; doi:10.1186/1472-6963-14-218)
Supplement: Additional file 3 — Intervention documentation. [file 1472-6963-14-218-S3.pdf]

Document	Category	Description
Presentation by consultant	Information about the intervention	PowerPoint presentation of the intervention and plan for implementation. March 2010.
Presentation by consultant	Information about the intervention	PowerPoint presentation with information and basis for discussion to be used at a planning day at the Section of Internal Medicine. May 2010.
Presentation by senior medical manager	Information about the intervention	PowerPoint presentation describing background and key measures relevant for the teamwork intervention. May 2010.
Handbook	Information about the intervention	Document describing the teamwork intervention and role descriptions for all professions. Used from May 2010.
Evaluation of teamwork	Information about the intervention	Internal evaluation of the teamwork intervention done by the ED change facilitator and presented to staff in November 2010.
Change facilitator	Information about the intervention	Role description and clear mandate for the change facilitator. This information was approved by the senior medical manager and sent from the change facilitator to all staff working at the ED in September 2010.
Project description VÖK 2010	Description from consultants	Formal document containing an overview of the implementation project.
Case 12	Description from consultants	Document describing the project; later used by the ED to apply for internal funding to finance the implementation of teamwork.
Project plan	Description from consultants	Overview of plan and method for the project.
Report on project status VÖK 2010	Description from consultants	Implementation plan. Detailed description of present status and detailed plan of the implementation. February 2011.
Checklist for teamwork 2010	Checklist	Checklist for teamwork used during the pilots in spring 2010.
Key teamwork behaviors	Checklist	"Flashcard" with key teamwork behaviors for all professions.
Checklist for debriefing meetings	Checklist	Checklist used at the daily debriefing meetings during fall 2010.
Physician specialized in emergency care	Role description	Role description for physicians specialized in emergency care. Parallel project that related to the teamwork intervention. Used during 2010-11.
Cardiology consultant	Role description	Description of the cardiology consultant's role in relation to teamwork. Used from September 2010.
Specialist	Role description	Detailed role description/checklist for the section specialist, March 2011. Observe that there is also a description of the specialist's role in the handbook from May 2010.
Week 34	ED weekly information sheet	Short info on updates regarding teamwork in 2010. This document is used only at the Section of Internal Medicine.
Week 36	ED weekly information sheet	Short info on updates regarding teamwork in 2010. This document is used only at the Section of Internal Medicine.
